# Testing measurement invariance in a conditional likelihood framework by considering multiple covariates simultaneously

**DOI:** 10.3758/s13428-024-02551-9

**Published:** 2025-01-08

**Authors:** Clemens Draxler, Andreas Kurz

**Affiliations:** 1 UMIT TIROL - Private University for Health Sciences and Technology, Eduard-Wallnöfer-Zentrum 1, 6060 Hall in Tirol, Austria; 2 Paris Lodron University Salzburg, Kapitelgasse 4-6, 5020 Salzburg, Austria

**Keywords:** Mixed logit model, Conditional maximum likelihood, Item parameter invariance, Rasch model

## Abstract

This article addresses the problem of measurement invariance in psychometrics. In particular, its focus is on the invariance assumption of item parameters in a class of models known as Rasch models. It suggests a mixed-effects or random intercept model for binary data together with a conditional likelihood approach of both estimating and testing the effects of multiple covariates simultaneously. The procedure can also be viewed as a multivariate multiple regression analysis which can be applied in longitudinal designs to investigate effects of covariates over time or different experimental conditions. This work also derives four statistical tests based on asymptotic theory and a parameter-free test suitable in small sample size scenarios. Finally, it outlines generalizations for categorical data in more than two categories. All procedures are illustrated on real-data examples from behavioral research and on a hypothetical data example related to clinical research in a longitudinal design.

## Introduction

This article addresses some basic problems in psychometrics. Its focus is on issues connected with statistical inference on measurement invariance. In general, the term refers to assuming the same measurement principles for different groups of persons or examinees in the population of interest. Specifically, in this work, it refers to testing the hypothesis of invariance of item parameters of the Rasch model (Fischer & Molenaar, 1995; Rasch, 1960) across multiple groups of persons in a conditional maximum likelihood (CML) framework (Andersen, 1970; Pfanzagl, 1993; Skrondal & Rabe-Hesketh, 2022). As such, it can also be viewed as a procedure for investigating differential item functioning (e.g., Holland & Wainer, 1993). One of the most frequently applied statistical tests based on asymptotic theory serving this purpose has been discussed by Andersen (1973). An overview of large sample tests based on CML has been given by Glas and Verhelst (1995). More recent developments have been discussed by, e.g., Draxler, Kurz, Gürer, and Nolte (2024), Draxler, Kurz, and Lemonte (2022), and Kreiner and Christensen (2002; 2011). Tests not based on asymptotic theory and particularly suited for small samples are suggested by Chen and Small (2005), Christensen and Kreiner (2010), Draxler and Dahm (2020), Draxler and Kurz (2021), Draxler and Zessin (2015), Ponocny (2001), and Verhelst (2008). Program packages are readily available, for instance, the R (R Core Team, 2022) packages eRm (Mair & Hatzinger, 2007; Mair et al., 2023) and tcl (Draxler & Kurz, 2023).

All of these tests consider only one covariate, for example, testing the equality of item parameters between two or more gender groups. In a typical application, when analyzing psychometric data or developing new educational or psychological tests, one is usually interested in more than one covariate, e.g., gender, age, ethnicity, etc. The usual approach then is to carry out a statistical test for each covariate separately. The drawback of it is that error probabilities of the first kind or type I error probabilities or the sizes of the tests accumulate. This means that the probability of wrongly rejecting the hypothesis of invariance in at least one of the (multiple) tests is greater than in each of the single tests and is often left uncontrolled (unless adjustments are made). In other words, one obtains an uncontrolled probability of falsely stating that at least one covariate (one or more) does have an effect or does violate the invariance assumption when none of the covariates do. For example, when five covariates are considered and five tests, each with a predetermined size of 0.05 are carried out, the probability that at least one of them results in an error of the first kind amounts to 0.226, provided the tests are independent (i.e., conditional on the result of any of the tests the type I prob. of any other test does not change). When covariates are correlated (when their true correlations are not 0) this number will be smaller depending on the sizes of the correlations. Simple adjustments of the type I error probabilities of the individual tests are based on the assumption of independence of the covariates. Hence, such procedures are too conservative when covariates correlate, but the true correlations are usually unknown, which prevents researchers from making appropriate and exact adjustments in practice. Corrections not based on the independence assumption are more appropriate in such cases, for instance, the Benjamini–Hochberg procedure (Benjamini & Hochberg, 1995).

This article proposes a solution by eliminating the need for multiple testing. It discusses an approach and a model that considers any desirable number of covariates (as long as parameters are identified) and allows both estimating and testing their effects simultaneously in a conditional likelihood framework. Thus, only one hypothesis test is needed whose size or probability of the error of the first kind can be predetermined and controlled at any level. The model is a generalization of the Rasch model and can be viewed as a mixed model with logit link function, i.e., a mixed logit model, since it considers random (persons or examinees) and fixed (items and covariates) effects. It can also be viewed as a multivariate multiple regression model for binary (or other categorical) data (Cox, 1958). It is multivariate since it considers multiple items (to which persons respond) and it considers multiple (more than one) covariates or predictors or explanatory variables that linearly affect the logits of item response probabilities. The model not only allows for binary or nominal covariates. It also considers linear effects of real-valued covariates. This model has already been discussed by Gürer and Draxler (2023) in the context of machine learning and penalizing techniques of conditional likelihood functions but without providing a respective hypothesis test.

Kelderman (1984) suggested a similar approach by reformulating the Rasch model as a log-linear model that considers additional effects of categorical or categorized covariates (with CML estimation and hypothesis testing procedures, i.e., likelihood-ratio tests). The approach also includes modeling local dependence between items. Such ideas may also be described by graphical models that define a recursive structure (which can also be viewed as a causal structure) between all the quantities involved along with respective conditional probability distributions. In this context, Kreiner and Christensen (2011) suggested so-called graphical log-linear Rasch models.

The remainder of this text is organized as follows. Sec. “Theoretical foundation” introduces the model and discusses the theoretical foundation and technical issues of the approach. Sec. “Statistical tests” derives four different test statistics based on asymptotic theory serving the present purpose. It also presents a parameter-free test that can be used in small sample scenarios. Sec. “Outline of generalizations” gives an outline on generalizations. Sec. “Data examples” provides real data examples and notes further applications in longitudinal designs. Sec. “Final remarks” gives a discussion and final remarks.

## Theoretical foundation

Consider a parametric family of probability distributions specified by a psychometric model and indexed by parameters taking values in parameter space $\Theta$ being an open subset of Euclidean space. Assume that the true unknown probability distribution generating the observations in a sample space (from which the data are sampled) is a member of that family. The observations are obtained by the binary responses, e.g., correct, or incorrect, of a number (sample) of persons or examinees to a number of items. Additionally, data on a number of covariates are collected. The psychometric model is given by

$$\begin{aligned} P(Y_{ij} = 1) =&\frac{\exp \big (\tau _i + \alpha _j + \sum _p \delta _{jp} x_{ip} \big )}{1 + \exp (\tau _i + \alpha _j + \sum _p \delta _{jp} x_{ip})},\\&i = 1,\dots ,n, \, j = 1,\dots ,k, \, p = 1,\dots ,q, \end{aligned}$$

where $Y_{ij} \in \{0, 1\}$ is the binary response of person *i* to item *j* and $\tau _i \in \mathbb {R}$ is a person parameter usually interpreted as an ability, proficiency, or attitude. The parameters $\alpha _j \in \mathbb {R}$ and $\delta _{jp} \in \mathbb {R}$ characterize item effects and are usually interpreted as easiness or attractiveness. The former represents a baseline parameter of the respective item or a general level of easiness of the item (i.e., when all covariate values are 0) and the latter a conditional effect of item *j* given covariate *p* (i.e., a slope parameter). The *x* quantities are the observed covariate values, i.e., $x_{ip}$ is the value observed for person *i* in respect of covariate *p*. Setting all the $\delta$ parameters equal to 0 (no covariate has an effect) yields the Rasch model as a special case with the $\alpha$ parameters as the item parameters. Assume that the *k* responses of every single person are independent, i.e., local independence, and the persons are drawn independently from a population of interest. Let the binary responses of all *n* persons in the sample to all *k* items be arranged in an $n \times k$ matrix denoted by $\varvec{Y}$, i.e., the response matrix. Then, by using matrix multiplication, the joint distribution of all these responses is obtained by

$$\begin{aligned} P(\varvec{Y} = \varvec{y})&= \prod _{i = 1}^{n} \prod _{j = 1}^{k} P(Y_{ij} = y_{ij})\\&= \underbrace{\frac{1}{\prod _i \prod _j \big (1 + \exp (\tau _i + \alpha _j + \sum _p \delta _{jp} x_{ip} \big )}}_{C(\varvec{\tau }, \varvec{\alpha }, \varvec{\delta })} \\&\qquad \exp \bigg (\sum _i \sum _j \big (y_{ij} (\tau _i + \alpha _j + \sum _p \delta _{jp} x_{ip}) \big ) \bigg )\\&= C(\varvec{\tau }, \varvec{\alpha }, \varvec{\delta }) \exp \big (\varvec{\tau }^{\top } \varvec{r} + \varvec{\alpha }^{\top } \varvec{s} + \varvec{\delta }^{\top } \varvec{t} \big ), \end{aligned}$$

where $\varvec{\tau }^{\top } = (\tau _1, \dots , \tau _n)$ denotes an $n \times 1$ matrix (i.e., a column vector of length *n*) containing as elements all person parameters, $\varvec{\alpha }^{\top } = (\alpha _1,\dots ,\alpha _k)$ a $k \times 1$ matrix of baseline parameters, and $\varvec{\delta }^{\top } = (\varvec{\delta }_1^{\top },\dots ,\varvec{\delta }_q^{\top })$ a $k q \times 1$ matrix of the effects of all *q* covariates on all *k* items, where $\varvec{\delta }_p^{\top } = (\delta _{1p}, \dots , \delta _{kp})$ contains the effects of a single covariate *p*. Both $\tau$ and $\alpha$ parameters are nuisances in the present problem. The only parameters of interest are the $\delta$ parameters. For identifiability let $\alpha _1 = 0, \delta _{1p} = 0 \hspace{0.1cm} \forall p$, i.e., one element of $\varvec{\alpha }$ and one of each $\varvec{\delta }_p$ is not free. Note that this is not a sufficient condition for the parameters to be identified and that it is an arbitrary choice. One may also use any other item as an anchor or set the sum of respective parameters over all *k* items to 0 or any other constant. It can immediately be seen from the factorization criterion that the statistics $\varvec{R}$ (with realization $\varvec{r}$), $\varvec{S}$ (with realization $\varvec{s}$), and $\varvec{T}$ (with realization $\varvec{t}$) are sufficient for the class of distributions. It is a member of a multiparameter exponential family with $(\varvec{\tau }, \varvec{\alpha }, \varvec{\delta }) \in \Theta \subseteq \mathbb {R}^{n+k(q + 1)}$ as its natural parameter space, i.e., the first factor is a normalizing constant (that does not depend on $\varvec{Y} = \varvec{y}$) and the second factor depends on the observations only through the sufficient statistics

$$ \varvec{R}^{\top } = (R_1, \dots , R_n), \, R_i = \sum _{j = 1}^k Y_{ij}, $$

$$ \varvec{S}^{\top } = (S_1, \dots , S_k), \, S_j = \sum _{i = 1}^n Y_{ij}, $$

$$ \varvec{T}^{\top } = (\varvec{T}_1^{\top }, \dots , \varvec{T}_q^{\top }), \, \varvec{T}_p^{\top } = (T_{1p}, \dots , T_{kp}), \, T_{jp} = \sum _{i = 1}^n Y_{ij} x_{ip}. $$

Thus, the $n \times 1$ matrix $\varvec{R}$ contains the row sums or the person scores of the response matrix $\varvec{Y}$, the $k \times 1$ matrix $\varvec{S}$ the column sums or item scores, and the $k q \times 1$ matrix $\varvec{T}$ weighted column sums, where the weights are given by the respective covariate values. Hence, further considerations can be restricted to the distributions of the sufficient statistics. The joint distribution of $\varvec{R}$, $\varvec{S}$, and $\varvec{T}$, the marginal distribution of $\varvec{R}$, and the conditional distribution of $\varvec{S}, \varvec{T}$ given $\varvec{R} = \varvec{r}$ are obtained by

$$\begin{aligned} P(\varvec{R} = \varvec{r}, \varvec{S} = \varvec{s}, \varvec{T} = \varvec{t}) = C(\varvec{\tau }, \varvec{\alpha }, \varvec{\delta }) \exp (\varvec{\tau }^{\top } \varvec{r} + \varvec{\alpha }^{\top } \varvec{s} + \varvec{\delta }^{\top } \varvec{t}) h(\varvec{r}, \varvec{s}, \varvec{t}), \end{aligned}$$

$$\begin{aligned} P(\varvec{R} = \varvec{r}) = C(\varvec{\tau }, \varvec{\alpha }, \varvec{\delta }) \exp (\varvec{\tau }^{\top } \varvec{r}) \prod _{i = 1}^n \gamma _{r_i} (\varvec{\alpha }, \varvec{\delta }, x_{i1}, \dots , x_{iq}), \end{aligned}$$

and

$$\begin{aligned} P(\varvec{S} = \varvec{s}, \varvec{T} = \varvec{t} \mid \varvec{R} = \varvec{r})&= \frac{P(\varvec{R} = \varvec{r}, \varvec{S} = \varvec{s}, \varvec{T} = \varvec{t})}{P(\varvec{R} = \varvec{r})}\\&= \frac{\exp (\varvec{\alpha }^{\top } \varvec{s} + \varvec{\delta }^{\top } \varvec{t})}{\prod _{i = 1}^n \gamma _{r_i} (\varvec{\alpha }, \varvec{\delta }, x_{i1}, \dots , x_{iq})} h(\varvec{s}, \varvec{t}, \varvec{r}), \end{aligned}$$

where $h(\varvec{r}, \varvec{s}, \varvec{t})$ is a combinatorial function denoting the number of potential $n \times k$ response matrices that yield $\varvec{R} = \varvec{r}$, $\varvec{S} = \varvec{s}$, and $\varvec{T} = \varvec{t}$. It can be ignored since it does not depend on any of the parameters. The function $\gamma _{r_i}$ denotes an elementary symmetric function of order $r_i$, where $r_i \in \{0, \dots , k\}$ denotes the score of person *i* which can be an integer from 0 to *k*. In the present problem, it is not only a function of all the item parameters (i.e., baseline and effect parameters) but also all the covariates. It is given by

$$\begin{aligned}&\gamma _0(\varvec{\alpha }, \varvec{\delta }, x_{i1}, \dots , x_{iq}) = 1\\&\gamma _1(\varvec{\alpha }, \varvec{\delta }, x_{i1}, \dots , x_{iq}) = \exp (\alpha _1 + \sum _p \delta _{1p} x_{ip}) + \dots + \exp (\alpha _k + \sum _p \delta _{kp} x_{ip})\\&\gamma _2(\varvec{\alpha }, \varvec{\delta }, x_{i1}, \dots , x_{iq}) = \exp \big (\alpha _1 + \alpha _2 + \sum _p (\delta _{1p} + \delta _{2p}) x_{ip} \big )\\&+ \dots + \exp \big (\alpha _{k - 1} + \alpha _k + \sum _p (\delta _{k - 1, p} + \delta _{kp}) x_{ip} \big )\\&\gamma _3(\varvec{\alpha }, \varvec{\delta }, x_{i1}, \dots , x_{iq}) = \exp \big (\alpha _1 + \alpha _2 + \alpha _3 + \sum _p (\delta _{1p} + \delta _{2p} + \delta _{3p}) x_{ip} \big )\\&+ \dots + \exp \big (\alpha _{k - 2} + \alpha _{k - 1} + \alpha _k + \sum _p (\delta _{k - 2, p} + \delta _{k - 1, p} + \delta _{kp}) x_{ip} \big )\\&\vdots \\&\gamma _{k - 1}(\varvec{\alpha }, \varvec{\delta }, x_{i1}, \dots , x_{iq}) = \exp \big (\alpha _1 + \dots + \alpha _{k - 1} + \sum _p (\delta _{1p} + \dots + \delta _{k -1, p}) x_{ip} \big )\\&+ \dots + \exp \big (\alpha _2 + \dots + \alpha _k + \sum _p (\delta _{2p} + \dots + \delta _{kp}) x_{ip} \big )\\&\gamma _k(\varvec{\alpha }, \varvec{\delta }, x_{i1}, \dots , x_{iq}) = \exp \big (\alpha _1 + \dots + \alpha _k + \sum _p (\delta _{1p} + \dots + \delta _{kp}) x_{ip} \big ). \end{aligned}$$

Thus, $\gamma _1$ is composed of a sum over all items, each summand being a function of the baseline and the *q* covariate effect parameters associated with the respective item (i.e., one $\alpha$ par. and one $\delta$ par. for each covariate), $\gamma _2$ is composed of a sum over all (potential) pairs of items, each summand being a function of the two baseline and the 2*q* covariate effect parameters associated with the respective pair (i.e., one $\alpha$ par. for each item and one $\delta$ par. for each item and each covariate), $\gamma _3$ is composed of a sum over all (potential) triples of items, each summand being a function of the three baseline and the 3*q* covariate effect parameters associated with the respective triple, etc.

The conditional distribution $P(\varvec{S} = \varvec{s}, \varvec{T} = \varvec{t} \mid \varvec{R} = \varvec{r})$ does not depend on the person parameters. Treating it as a function of the remaining parameters and taking the logarithm yields the conditional log-likelihood

$$\begin{aligned} \ell (\varvec{\alpha }, \varvec{\delta }) = \varvec{\alpha }^{\top } \varvec{s} + \varvec{\delta }^{\top } \varvec{t} - \sum _{i = 1}^n \log \gamma _{r_i} (\varvec{\alpha }, \varvec{\delta }, x_{i1}, \dots , x_{iq}), \end{aligned}$$

where the additive constant $\log h(\varvec{s}, \varvec{t}, \varvec{r})$ is omitted.

From general likelihood theory and exponential families as well as particular results for the conditional likelihood case (Andersen, 1970; Pfanzagl, 1993), one readily obtains the score function, the Fisher information matrix, estimates of the parameters, and their properties. The vector-valued score function denoted by $\varvec{D}$ is given by the first-order partial derivatives of $\ell (\varvec{\alpha }, \varvec{\delta })$ with respect to all free $\alpha$ and $\delta$ parameters. Note that the first $\alpha$ parameter and the first $\delta$ parameter for each covariate have been set to 0 for identifiability. Thus, it has only length $(k - 1)(q + 1)$. It is a function of the last $k - 1$ elements of $\varvec{s}$ and every $\varvec{t}_p$ as well as the free $\alpha$ and $\delta$ parameters. It is simply given by the differences of the observed and expected values of the sufficient statistics $\varvec{S}$ and $\varvec{T}$ conditional on $\varvec{R} = \varvec{r}$ which holds generally for exponential families. The Fisher information matrix denoted by $\varvec{F}(\varvec{\alpha }, \varvec{\delta })$ can be obtained from the second-order partial derivatives of $\ell (\varvec{\alpha }, \varvec{\delta })$ with respect to all free parameters. Details and computational issues on the information matrix and score function are given in Appendix A.

The CML estimate of $(\varvec{\alpha }, \varvec{\delta })$ is defined by

$$ \big (\widehat{\varvec{\alpha }}, \widehat{\varvec{\delta }} \big ) := \underset{(\varvec{\alpha }, \varvec{\delta }) \in \mathbb {R}^{k(q + 1)}}{\arg \max } \hspace{0.2cm} \ell (\varvec{\alpha }, \varvec{\delta }) $$

which is obtained by solving $\varvec{D} = \varvec{0}_{(k - 1)(q + 1)}$ for the $\alpha$ and $\delta$ parameters. An R code is provided as supplementary material in an online repository which uses a numerical procedure known as the Broyden–Fletcher–Goldfarb–Shanno (BFGS) algorithm (Broyden, 1970; Fletcher, 1970; Goldfarb, 1970; Shanno, 1970). In order to obtain the usual asymptotic properties of maximum likelihood estimates a mild regularity condition has to be considered in the CML case (Andersen, 1970; Pfanzagl, 1993), i.e., the values of the person parameters ($\tau$ parameters) must not be too extreme. Then, it holds that

$$ (\widehat{\varvec{\alpha }}_*, \widehat{\varvec{\delta }}_*) \xrightarrow {P} (\varvec{\alpha }_*, \varvec{\delta }_*) $$

and

$$ \sqrt{n} \Big (\big (\widehat{\varvec{\alpha }}_*, \widehat{\varvec{\delta }}_* \big ) - (\varvec{\alpha }_*, \varvec{\delta }_*) \Big ) \xrightarrow {D} N \big ( \varvec{0}_{(k - 1)(q + 1)}, \varvec{F}^{-1}(\varvec{\alpha }, \varvec{\delta }) \big ) $$

when the number of persons $n \rightarrow \infty$, where $\varvec{F}^{-1}(\varvec{\alpha }, \varvec{\delta })$ is the asymptotic covariance matrix of the CML estimate. Note that the notation $\varvec{\alpha }_*, \varvec{\delta }_*$ is used to indicate that the respective vector contains only the free parameters. Note also that the responses of persons with a score (i.e., row sum in the response matrix $\varvec{Y}$) of 0 and *k* are completely uninformative. They have to be excluded.

Finally, a brief remark on identifiability of parameters. A parameter is obviously not identified or estimable when the conditional distribution given the observed value of its sufficient statistic is only 0 or 1, i.e., when exactly one response pattern is associated with the respective value of the sufficient statistic. Such data are completely uninformative. Necessary and sufficient conditions for all the parameters to be identified have only been given for the Rasch model thus far (Fischer, 1981).

## Statistical tests

Let the following two subclasses of distributions or, equivalently, parameter spaces be defined by

$$ \Theta _1 = \{(\varvec{\tau }, \varvec{\alpha }, \varvec{\delta }) \mid (\varvec{\tau }, \varvec{\alpha }, \varvec{\delta }) \in \Theta , \varvec{\delta }= \varvec{0} \} $$

and

$$ \Theta _2 = \{(\varvec{\tau }, \varvec{\alpha }, \varvec{\delta }) \mid (\varvec{\tau }, \varvec{\alpha }, \varvec{\delta }) \in \Theta , \varvec{\delta }\ne \varvec{0} \}, $$

$\Theta _1 \cup \Theta _2 = \Theta$. Of interest is the hypothesis that the true unknown distribution (or the true parameters) satisfies

$$ (\varvec{\tau }, \varvec{\alpha }, \varvec{\delta }) \in \Theta _1 $$

(as assumed by the Rasch model) against the alternative

$$ (\varvec{\tau }, \varvec{\alpha }, \varvec{\delta }) \in \Theta _2. $$

The latter represents the scenario that at least one of the *q* covariates has an effect on at least one item.

Four different test statistics derived from the properties of the CML estimates based on asymptotic theory may be used. Let $\widehat{\varvec{\alpha }}_0$ denote the restricted CML estimate of $\varvec{\alpha }$, i.e., the argument of the maximum of $\ell (\varvec{\alpha }, \varvec{\delta })$ given $\varvec{\delta }= \varvec{0}$. Thus, the vector $\widehat{\varvec{\alpha }}_0$ contains the estimates of the item parameters of the simple Rasch model. Then, one obtains a likelihood-ratio test statistic (Neyman & Pearson, 1928; Wilks, 1938) by evaluating $\ell (\varvec{\alpha }, \varvec{\delta })$ at both the restricted and the unrestricted estimates:

$$ LR = -2 \Big (\ell \big (\widehat{\varvec{\alpha }}_0, \varvec{\delta }= \varvec{0} \big ) - \ell \big (\widehat{\varvec{\alpha }}, \widehat{\varvec{\delta }} \big ) \Big ). $$

This is simply a generalization of the well-known Andersen likelihood-ratio test (Andersen, 1973) for the case of more than one covariate and real-valued covariates. A Rao score test statistic (Rao, 1948) can be obtained by

$$ RS = \varvec{D}^{\top }(\widehat{\varvec{\alpha }}_0, \varvec{\delta }= \varvec{0}) \varvec{F}^{-1}(\widehat{\varvec{\alpha }}_0, \varvec{\delta }= \varvec{0}) \varvec{D}(\widehat{\varvec{\alpha }}_0, \varvec{\delta }= \varvec{0}), $$

where both the score function $\varvec{D}$ and the information matrix $\varvec{F}$ are evaluated only at the restricted estimates. Thus, $\varvec{\delta }$ need not be estimated at all. This is quite a remarkable feature of the score test, which sets it apart from others. Note that the score test is also called the Lagrange multiplier test (mainly in econometrics) since the test statistic can be expressed in terms of Lagrange multipliers (Silvey, 1959). A Wald test statistic (Wald, 1943) is given by

$$ W = \widehat{\varvec{\delta }}_*^{\top } \varvec{\Sigma }^{-1} \big ( \widehat{\varvec{\alpha }}, \widehat{\varvec{\delta }} \big ) \widehat{\varvec{\delta }}_*, $$

where the notation $\widehat{\varvec{\delta }}_*$ (again) is used to indicate that the respective vector is assumed to contain only the estimates of the free $\delta$ parameters. The matrix $\varvec{\Sigma }$ denotes the covariance matrix of $\widehat{\varvec{\delta }}_*$. It is obtained by omitting the first $k - 1$ rows and columns (that refer to $\varvec{\alpha }_*$ which is not of interest) of the complete covariance matrix $\varvec{F}^{-1}$ evaluated at the unrestricted estimates $(\widehat{\varvec{\alpha }}, \widehat{\varvec{\delta }} \big )$. Finally, a gradient test statistic (Lemonte, 2016; Terrell, 2002) is obtained by

$$ G = \varvec{D}_*^{\top } (\widehat{\varvec{\alpha }}_0, \varvec{\delta }= \varvec{0}) \widehat{\varvec{\delta }}_*, $$

where $\varvec{D}_*^{\top } = (\varvec{D}_1^{\top }, \dots , \varvec{D}_q^{\top })$ is evaluated at the restricted estimates. All four test statistics have a common limiting distribution when $(\varvec{\tau }, \varvec{\alpha }, \varvec{\delta }) \in \Theta _1$ holds true and $n \rightarrow \infty$. It is the central $\chi ^2$ distribution with $df = (k - 1)q$, i.e., $k - 1$ free $\delta$ parameters per covariate.

Note that the gradient test is a relatively recent development in the theory of statistics. It is, thus, still little known in psychological and educational communities. It has only been discussed in the context of psychometric problems in three articles. Draxler et al. (2022) and Draxler et al. (2024) discuss it in a conditional likelihood and Zimmer, Draxler, and Debalak (2023) in a marginal likelihood framework. The test statistic can be derived from a combination of the Rao score and Wald test statistics. An obvious computational advantage of it is that it does not depend on an information matrix.

### A parameter-free test

This approach considers conditioning on the observed values of the sufficient statistics for all nuisance parameters ($\varvec{\tau }$ and $\varvec{\alpha }$) which are given by the row and column sums of the response matrix. It considers the conditional distribution of the sufficient statistics for the $\delta$ parameters being the only parameters of interest (in the present problem). The marginal distribution of the vectors of sufficient statistics for all nuisance parameters is given by

$$ P(\varvec{R} = \varvec{r}, \varvec{S} = \varvec{s}) = C(\varvec{\tau }, \varvec{\alpha }, \varvec{\delta }) \exp (\varvec{\tau }^{\top } \varvec{r} + \varvec{\alpha }^{\top } \varvec{s}) \sum _\Omega \exp (\varvec{\delta }^{\top } \varvec{t}). $$

The conditional distribution is then obtained by

$$ P(\varvec{T} = \varvec{t} \mid \varvec{R} = \varvec{r}, \varvec{S} = \varvec{s}) = \frac{P(\varvec{R} = \varvec{r}, \varvec{S} = \varvec{s}, \varvec{T} = \varvec{t})}{P(\varvec{R} = \varvec{r}, \varvec{S} = \varvec{s})} = \frac{\exp (\varvec{\delta }^{\top } \varvec{t})}{\sum _\Omega \exp (\varvec{\delta }^{\top } \varvec{t})}, $$

where $\Omega$ denotes the restricted sample space (that follows from the conditioning) consisting of all potential $n \times k$ response matrices yielding row and column sums $\varvec{R} = \varvec{r}$ and $\varvec{S} = \varvec{s}$ (i.e., yielding the same row and column sums as the observed response matrix). The summations on the right sides of the two equations have accordingly to be taken over all elements of $\Omega$. Note that this is a summation over all potential values of the vector-valued statistic $\varvec{T}$, i.e., matrices in the sample space can yield different values of $\varvec{T}$, where the range of possible values for each element of the vector $\varvec{T}$ is determined by the condition $\varvec{R} = \varvec{r}$ and $\varvec{S} = \varvec{s}$. Thus, it is a normalizing constant ensuring that the respective probabilities sum up to 1. Again, treating this conditional distribution as a function of the only remaining parameter vector and taking the logarithm yields a conditional log-likelihood function

$$ \ell (\varvec{\delta }) = \varvec{\delta }^{\top } \varvec{t} - \log \sum _\Omega \exp (\varvec{\delta }^{\top } \varvec{t}). $$

Since this conditional distribution is also a multiparameter exponential family (as can immediately be seen) all well-known results of likelihood and asymptotic theory in respect of properties of the estimates and the distribution of the four $\chi ^2$ test statistics hold true and are principally applicable in this case too. Technical details are given in Appendix B.

Practically, applying asymptotic theory does not make much sense in this case since the enumeration of all potential matrices, i.e., all elements contained in $\Omega$, is computationally infeasible for cases with usual numbers of persons and items. Miller and Harrison (2013) solved the complicated combinatorial problem of determining the exact number of matrices, i.e., the cardinality of $\Omega$, by deriving a recursive algorithm based on graph theory but computing it is nevertheless very intensive and this number itself does not suffice to determine the exact conditional distribution of the statistic $\varvec{T}$. One needs to enumerate all matrices. Fortunately, algorithms designed to sample each element of $\Omega$ with approximately the same probability (i.e., to obtain a simple random sample) can be applied to approximate the conditional distribution of $\varvec{T}$. Verhelst (2008), for instance, suggests a Markov chain Monte Carlo algorithm whose stationary distribution is given by a discrete uniform distribution of the elements of $\Omega$. Miller and Harrison (2013) suggest an exact sampling approach, i.e., each element is selected with exactly the same probability. Given $\varvec{\delta }= \varvec{0}$ (i.e., the hypothesis of interest) and having obtained a simple random sample of matrices (by applying one of these algorithms) the exact conditional distribution of $\varvec{T}$ can be arbitrarily well approximated by simply considering the distribution of relative frequencies with which the different values of every element of $\varvec{T}$ are observed in the random sample (of all matrices drawn). Verhelst’s algorithm seems to be the most efficient choice in respect of computing time so far (e.g., Draxler & Nolte, 2018) and it is readily available as an R package called RaschSampler (Verhelst et al., 2007).

An obvious choice of a test statistic is the Rao score since the score function and information matrix (given in Appendix B) have to be evaluated only at $\varvec{\delta }= \varvec{0}$. As such it can be viewed as a parameter-free test. Since the score function is simply given by the difference of observed and expected value of $\varvec{T}$ and since the expected value is a constant the exact distribution of the score function is the same as the conditional distribution of $\varvec{T}$ itself. Accordingly, it can be arbitrarily well approximated from a simple random sample of matrices drawn. One simply computes the mean for each element of $\varvec{T}$ of all matrices drawn as an approximation or estimate of the respective expected value and considers the difference to the observed value in every one of the matrices drawn. Similarly, one obtains an approximation of the information matrix by simply computing the sample covariances from all the matrices drawn. Henceforth, one obtains a value of the Rao score test statistic for each matrix drawn and the *p* value for the observed response matrix is obtained from that distribution.

This test can of course be recommended in scenarios of small sample sizes when a poor approximation (of the exact distribution) by $\chi ^2$ is to be expected.

### Power function of the tests

The power of a test is a function of the unknown parameters given its size (type I error probability) and the ample size. In the multiparameter case, it seems to be convenient and practical to use a function of all the unknown parameters. It is typically called an effect measure (Cohen, 1988). In the present problem, it is easily obtained by dividing the respective $\chi ^2$ test statistic by the informative sample size that excludes persons with a score of 0 and *k* (Draxler, 2010; Draxler & Alexandrowicz, 2015). This yields a sort of pseudo $R^2$, which can be interpreted as the proportion of variance explained by the covariates considered.

**Fig. 1:**
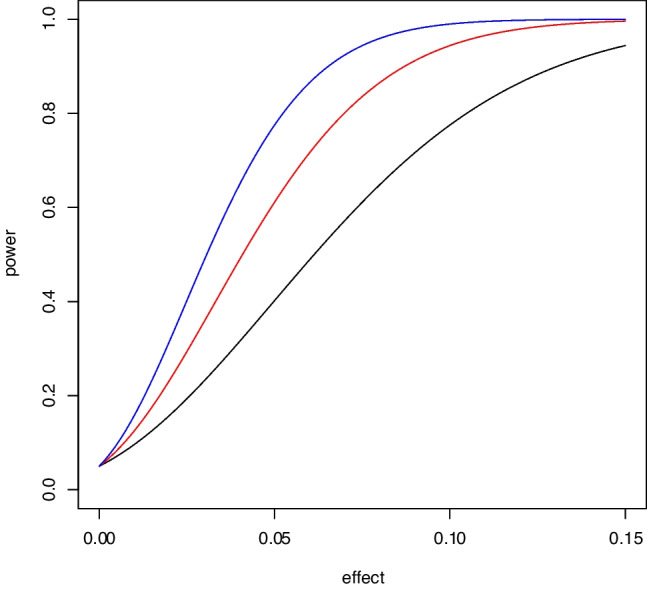
Power curves given a type I error probability of 0.05 and $df = 20$. The *black line* represents the case of an informative sample size of 200, the *red line* of 300, and the *blue line* of 400

The Rasch model does not allow any differences in the logits of response probabilities between persons with different covariate values (only between persons with different person parameters). Thus, the effect (of all the covariates) is 0. In this case, the power of the tests equals their given size (i.e., type I error prob.). Figure 1 shows examples of power curves for different informative sample sizes given a type I error probability of 0.05 and given the number of degrees of freedom of the test is 20. Note that the case $df = 20$ can be obtained in different scenarios regarding the numbers of covariates and items, for instance, when $q = 1 \, , k = 21$ or $q = 2 \, , k = 11$ or $q = 4 \, , k = 6$. In case of an informative sample of size 300, for example, the power yields 0.61 given an effect of 0.05 (one- twentieth of explained variance by the covariates), and it yields 0.94 given an effect of 0.1 (one-tenth of explained variance). Thus, when the true effect is 0.05 or greater the Rasch model is rejected with a probability of at least 0.61. When the true effect is 0.1 or greater the Rasch model is rejected with a probability of at least 0.94. Note that the validity (and accuracy) of all these considerations depend on the $\chi ^2$ approximation of the distribution of the respective test statistics, i.e., the non-central $\chi ^2$ with $df = (k - 1)q$ and non-centrality parameter given by the product of the effect and informative sample size (in case of an effect of 0 it reduces to the central $\chi ^2$, of course) (Draxler & Alexandrowicz, 2015). If it is poor, the power function may also be inaccurate.

## Outline of generalizations

Given the theoretical foundation of the approach of testing invariance of item parameters discussed in this work, a generalization to models that consider item responses in more than two nominal or ordinal categories is obvious and straightforward. It is also of great practical interest. For instance, the partial credit model (Masters, 1982) that considers ordinal responses in potentially more than two categories is one of the most popular and most frequently applied models in psychometric problems. In general, the following multiparameter exponential family may principally be considered:

$$ P(\varvec{Y} = \varvec{y}) = C(\varvec{\vartheta }, \varvec{\theta }) \exp \big (\varvec{\vartheta }^{\top } \varvec{u}_1 + \varvec{\theta }^{\top } \varvec{u}_2 \big ) h(\varvec{y}), $$

where $(\varvec{\vartheta }, \varvec{\theta }) \in \Theta$ with $\varvec{\vartheta }$ as a vector of nuisance parameters and $\varvec{\theta }$ a vector of parameters of interest. The former typically represents characteristics of persons and the latter is a vector of parameters that represent characteristics of items and their response categories as well as the effects of covariates. The vectors $\varvec{u}_1$ and $\varvec{u}_2$ are the observed values of their respective sufficient statistics, which are functions of the data $\varvec{y}$ (being nonnegative integers representing the responses of the persons to the items) and the covariate values.

The derivation of the conditional distribution given the observed values of the sufficient statistics for the nuisance parameters $P(\varvec{U}_2 = \varvec{u}_2 \mid \varvec{U}_1 = \varvec{u}_1)$, the respective conditional likelihood function, CML estimates, and their properties is straightforward for the given class of models. Statistical tests based on asymptotic theory are obtained along the same lines as discussed in Sec. “Statistical tests”. Their derivation follows the same principles.

Further extensions may also be interesting and suitable, i.e., modeling local dependence and considering other than linear effects of the covariates. The former has already been discussed in the framework of log-linear and graphical log-linear Rasch models. In respect of the latter, relaxing the linearity assumption and considering any monotone or non-monotone dependence of the logits of item responses on real-valued covariates is certainly of interest when the models are applied in a wider experimental context and a longitudinal design to investigate the type of effects of the covariates or predictors (as illustrated in Sec. “Example 3” in Example 3). It may be less of interest in the psychometric context, i.e., when the objective is to develop a measurement invariant and DIF-free instrument. Using splines as regression functions defined piecewise by polynomials could be a promising approach.

Furthermore, one may also consider a multidimensional model that assumes a vector-valued individual person parameter. Such an extension should be straightforward in a scenario where every item is assigned to only one dimension (or factor). Then, the number of correct responses to all the items assigned to the same dimension should be a sufficient statistic for the respective element of the vector-valued person parameter (i.e., the person parameter referring to the respective dimension), and conditional inference should apply. Other multidimensional extensions may be more complicated, for instance, when items are assigned to more than one factor simultaneously.

## Data examples

This section is aimed at illustrating the application of the estimation and testing procedures on a number of real-data and hypothetical examples and the interpretation of the results.

### Example 1

The first example of data can be found in the R package sirt (Robitzsch, 2022). The files are called data.pisaMath.rda and data.pisaRead.rda. The former contains binary responses of 565 students to 11 mathematics items and the latter binary responses of 623 students to 12 reading items. Both consider three covariates: gender, which is binary, a real-valued index of the socio-economic status (of the students’ families) called hisei, and migration background abbreviated by migra, which is again a binary covariate. Results of estimates and respective standard errors are shown in Table 1. As can be seen standard errors are smaller for the estimates referring to the real-valued covariate hisei. The largest standard errors are obtained for the parameters referring to covariate migra, i.e., this covariate provides little information since the proportion of students with migration background in the sample is quite low. Appendix C provides additional tables with *Z* test statistics for each single parameter, i.e., testing the hypothesis that the true value of the respective parameter is 0 against the alternative of $\ne 0$. These are simply obtained by dividing the respective estimate by its standard error, i.e., the square of it yields the Wald test statistic with $df = 1$. Note that item 1 is used as an anchor or baseline. If item 1 was affected by DIF or, precisely speaking, the true slope parameter (of a covariate for item 1) was not 0 the estimates of the slope parameters of the other items would simply reflect deviations from that certain amount of DIF of item 1. Thus, estimates near 0 would confirm the presence (not the absence) of DIF for those items. In the practice of psychometric analysis, a sum normalization of item parameters may be more preferable.

**Table 1:** Conditional maximum likelihood estimates with standard errors in parentheses for both data examples

	Math data				Reading data			
Item	Baseline	Gender	Hisei	Migra	Baseline	Gender	Hisei	Migra
2	$-0.134$	0.142	$-0.179$	$-0.089$	$-1.096$	$-0.482$	$-0.157$	0.333
	(0.205)	(0.278)	(0.137)	(0.538)	(0.252)	(0.368)	(0.194)	(0.550)
3	$-0.881$	$-0.141$	$-0.276$	$-0.764$	$-5.428$	$-0.752$	$-0.221$	0.112
	(0.209)	(0.295)	(0.146)	(0.739)	(0.356)	(0.476)	(0.241)	(0.789)
4	1.348	0.368	$-0.289$	0.267	2.194	0.244	0.047	0.445
	(0.222)	(0.294)	(0.145)	(0.498)	(0.452)	(0.761)	(0.377)	(0.927)
5	0.275	0.445	$-0.090$	0.151	$-0.199$	$-0.710$	$-0.103$	0.879
	(0.206)	(0.277)	(0.137)	(0.498)	(0.271)	(0.390)	(0.205)	(0.597)
6	1.208	0.286	0.041	0.688	0.107	$-0.478$	0.388	0.144
	(0.221)	(0.292)	(0.147)	(0.505)	(0.282)	(0.405)	(0.218)	(0.573)
7	0.005	0.502	$-0.339$	0.733	$-1.693$	0.641	$-0.324$	0.002
	(0.203)	(0.274)	(0.135)	(0.492)	(0.246)	(0.360)	(0.189)	(0.543)
8	$-0.006$	0.612	$-0.108$	0.616	$-0.016$	$-0.368$	$-0.210$	$-0.087$
	(0.205)	(0.275)	(0.136)	(0.492)	(0.275)	(0.403)	(0.212)	(0.577)
9	$-0.265$	1.375	0.153	0.068	$-1.846$	$-0.847$	$-0.152$	0.329
	(0.207)	(0.280)	(0.140)	(0.502)	(0.246)	(0.359)	(0.189)	(0.542)
10	0.092	0.755	$-0.137$	0.433	$-2.361$	$-0.940$	$-0.359$	$-0.016$
	(0.205)	(0.275)	(0.137)	(0.491)	(0.246)	(0.359)	(0.188)	(0.559)
11	$-0.625$	1.229	0.039	0.702	$-3.847$	$-1.198$	$-0.529$	$-0.132$
	(0.207)	(0.277)	(0.138)	(0.496)	(0.270)	(0.394)	(0.204)	(0.659)
12	$-$	$-$	$-$	$-$	$-0.412$	$-1.174$	0.015	0.264
	$-$	$-$	$-$	$-$	(0.265)	(0.376)	(0.198)	(0.556)

Note. Item 1 omitted. Its parameters are set to 0 for identifiability

The results of the four $\chi ^2$ tests and their observed effects (observed test statistic divided by informative sample size) as well as the power obtained for the respective observed effect (post hoc power) are shown in Table 2.

**Table 2:** Results of four $\chi ^2$ tests for both data examples

	*LR*	*RS*	*W*	*G*
Math data				
test statistic	89.971	87.662	85.572	91.857
*df*	30	30	30	30
*p* value	$< 0.001$	$< 0.001$	$< 0.001$	$< 0.001$
observed effect	0.170	0.165	0.162	0.173
power	$> 0.999$	$> 0.999$	$> 0.999$	$> 0.999$
Reading data				
Test statistic	53.318	52.255	51.273	54.150
*df*	33	33	33	33
*p* value	0.014	0.018	0.022	0.012
Observed effect	0.088	0.086	0.084	0.089
Power	0.996	0.996	0.995	0.997

Note. Power values shown refer to the power of the tests for the observed effect given a type I error probability of 0.05

The parameter-free Rao score test based on an approximate simple random sample of matrices (i.e., each one drawn with approx. the same prob.) of size 8191 using the R package RaschSampler (the max. number allowed by the package) yields for the math data $RS = 87.581$, *p* value $< 0.001$ and for the reading data $RS = 51.795$, *p* value $= 0.019$. The procedure of drawing random samples of matrices and computing *RS* has been replicated a number of times (i.e., about 20 times) to check the accuracy and reliability of the approximation. The results do not differ substantially and are, thus, quite stable. Furthermore, it can be observed that practically relevant percentiles like the 90th, 95th, and 99th of the distribution of *RS* obtained from the random samples drawn do barely deviate from the respective percentiles of the $\chi ^2$ distribution with $df = (k - 1)q$. Thus, the parameter-free Rao score test yields reliable results.

One may also make the following additional comparisons of models by using information criteria like *AIC* (Akaike, 1974) and *BIC* (Schwarz, 1978) as well as statistical tests. Table 3 shows *AIC* and *BIC* values computed for four models and both data examples: model 1 is the Rasch model that considers no covariate, model 2 considers only gender as a covariate, model 3 considers gender and hisei as covariates, and model 4 considers all three covariates available gender, hisei, and migra. According to *AIC* model 3 is most appropriate (in relation to the other choices) for both math and reading data, i.e., gender and hisei seem to have a considerable effect on the item responses, whereas migra does not. *BIC* prefers model 2 (considering only gender) for both math and reading data. *BIC* is known to be conservative. It prefers simple models with less parameters since it penalizes much more for additional parameters than *AIC*.

**Table 3:** Information criteria for four models and both data examples

	Math data		Reading data	
Model	*AIC*	*BIC*	*AIC*	*BIC*
1	4853.5	4896.2	3534.6	3583.2
2	4799.5	4842.3	3514.8	3563.3
3	4795.1	4880.6	3508.2	3605.2
4	4803.5	4931.7	3525.3	3670.9

**Table 4:** Conditional maximum likelihood estimates with standard errors in parentheses for Example 2

Item	Base	Gender	Age	Mean grade
2	$-$1.694 (3.731)	0.512 (1.203)	0.060 (0.136)	−0.430 (0.805)
3	0.775 (3.405)	0.496 (1.185)	$-$0.037 (0.115)	−0.624 (0.789)
4	$-$0.569 (3.207)	0.163 (1.093)	$-$0.040 (0.109)	$-$0.517 (0.750)
5	$-$2.920 (3.172)	1.261 (1.075)	0.011 (0.109)	−0.651 (0.739)
6	$-$2.454 (3.274)	1.253 (1.109)	0.035 (0.114)	−0.546 (0.753)
7	$-$1.664 (3.163)	0.655 (1.072)	$-$0.048 (0.108)	−0.589 (0.739)
8	0.260 (3.443)	0.185 (1.168)	0.000 (0.119)	−0.733 (0.778)
9	$-$4.098 (3.686)	0.143 (1.145)	0.127 (0.138)	−0.152 (0.787)
10	$-$2.177 (3.165)	0.827 (1.074)	$-$0.046 (0.108)	−0.340 (0.741)

Note. Item 1 omitted. Its parameters are set to 0 for identifiability

Likelihood-ratio tests are also readily applicable for comparing models. For the math data one obtains for comparing models 3 and 4, i.e., testing the hypothesis that the slope parameters of covariate migra are all 0, $LR = 11.636, \, df = 10$, *p* value $= 0.31$. The comparison of models 2 and 3, i.e., testing the hypothesis that the slope parameters of covariate hisei are all 0, yields $LR = 24.39, \, df = 10$, *p* value $= 0.007$, and comparing models 1 (Rasch model) and 3, i.e., testing the hypothesis that the slope parameters of both gender and hisei are all 0, yields $LR = 78.33, \, df = 20$, *p* value $< 0.001$. Hence, given sizes of the tests typically used in practice, in all three tests model 3 has to be accepted or chosen which is in accordance with the results of the *AIC*. Similar results are obtained for the reading data.

Note that comparisons of models using likelihood-ratio tests are always feasible and practical as long as models are nested. In this example, this is the case and, thus, every model can be compared to any other (more general) model.

### Example 2

The second example refers to the admission procedure for secondary-level general education in Austria (i.e., Verbund Mitte Austria). Pfaffel and Ecker (2023) recently presented the main elements of the procedure as well as initial results on predictive validity. Data stem from examinees or participants that were accepted into the teacher training program at a university in Austria. This example presents only an analysis of one part of the data which refers to measuring social understanding. The data contain 426 binary responses to ten items and consider four covariates: gender which is binary, age, and the mean grade (1 is the best and 5 the worst) of the participants’ first year of study.

**Table 5:** Results of four $\chi ^2$ tests for Example 2

	LR	RS	W	G
Test statistic	29.966	28.110	26.843	31.734
*df*	27	27	27	27
*p* value	0.316	0.405	0.472	0.242
Observed effect	0.108	0.101	0.097	0.114
Power	0.910	0.885	0.865	0.929

Note. Power values shown refer to the power of the tests for the observed effect given a type I error probability of 0.05

Tables 4, 5, and 11 in Appendix C show results. Results of estimates and respective standard errors are shown in Table 4. Standard errors are generally quite large since the informative sample size is only 278, i.e., 148 persons responded correctly to all items and are, thus, completely uninformative. The negative estimates (of the slope parameters) of covariate mean grade imply that the items get harder compared to item 1 with increasing mean grades (i.e., worse grades) of the persons (but standard errors are all larger than the negative deviation of the estimates from 0). Table 5 shows the results of the $\chi ^2$ tests, i.e., testing the hypothesis that the slope parameters of all covariates are 0.

### Example 3

An application that goes beyond typical psychometric problems referring to a wider empirical and experimental context may be the following. Since the approach discussed in this work uses a random intercept model or mixed-effects model (for binary data) with the person parameters as random effects it is perfectly suited for modeling responses of persons in longitudinal designs and, thus, investigating the effects of covariates over time (i.e., a number of discrete time points). In clinical research, for instance, one may observe diseases and symptoms of patients repeatedly at particular points in time and is typically interested in covariates like gender, age, treatment conditions, drug dosages, etc. The theoretical groundwork of such a regression analysis of binary sequences (together with conditional distributions and tests) dates back to the work of Cox (1958).

**Table 6:** Conditional maximum likelihood estimates with standard errors (SE) for Example 3

	Spring	Summer	SE	Autumn	SE	Winter	SE
Baseline	0	$-1.268$	0.886	0.383	0.932	2.231	1.134
Gender	0	$-0.396$	0.256	$-0.540$	0.273	$-0.406$	0.324
Age	0	0.023	0.020	0.028	0.020	0.009	0.025
Treatment	0	0.070	0.255	$-0.779$	0.273	$-0.703$	0.328

Note. Spring season is used as a baseline. The respective parameters are set to 0

**Table 7:** *Z*-test statistics with respective two-sided *p* values for each single free parameter of Example 3, i.e., parameter referring to spring omitted

	Summer	*p* value	Autumn	*p* value	Winter	*p* value
Baseline	$-1.431$	0.153	0.411	0.681	1.967	0.049
Gender	$-1.545$	0.122	$-1.983$	0.047	$-1.253$	0.210
Age	1.160	0.246	1.345	0.179	0.366	0.715
Treatment	0.274	0.784	$-2.852$	0.004	$-2.142$	0.032

As an example, consider investigating seasonal affective disorder or seasonal depression. Participants or patients respond in each of the four seasons of the year. In the simplest case, it is only a binary response, i.e., depression is present or not. Thus, one obtains four binary responses from every patient, i.e., one per season. The covariates considered are: gender (binary), age in years, and treatment condition (binary), i.e., half of the sample of patients receives a treatment, the other half does not. Hypothetical data of $n = 600$ patients are generated assuming the following: The baseline parameters ($\alpha$ parameters) are chosen as to characterize a realistic scenario that the prevalence of seasonal depression is generally lower in spring and summer seasons and higher in autumn and winter. The slope parameters ($\delta$ parameters) of the covariates gender and age are chosen to be 0 (i.e., no effect of gender and age on seasonal depression), whereas the choice of the slope or effect parameters of the treatment condition reflects a reduction of the probabilities of observing depression in the autumn and winter seasons in the treatment group. The spring season is selected to be time point 1 and is, thus, considered as a baseline, i.e., $\alpha$ and $\delta$ parameters referring to time point 1 are set to 0 (for identifiability). Thus, the parameters referring to the other time points or seasons express their effects relative to time point 1 (spring). Tables 6, 7, and 8 show results. It can immediately be seen that covariate treatment has an effect in the autumn and winter seasons. The respective estimates are distinctly smaller than 0 indicating a decline of prevalence of depression in the treatment group (as an effect of the treatment). From the estimates of the baseline parameters ($\alpha$ parameters) it can also be seen that the prevalence rate generally drops in summer (compared to spring) and increases in autumn and winter.

**Table 8:** Results of four $\chi ^2$ tests for Example 3

	Test statistic	*df*	*p* value	Obs. effect	Power
*LR*	19.173	9	0.024	0.041	0.887
*RS*	18.979	9	0.025	0.041	0.887
*W*	18.583	9	0.029	0.040	0.878
*G*	19.375	9	0.022	0.041	0.887

Note. Power values shown refer to the power of the tests for the observed effect given a type I error probability of 0.05

When comparing different models using information criteria, one yields the following results. According to both *AIC* and *BIC* the data support the model considering the treatment group as the only covariate (relative to the other choices or models), i.e., it is the only covariate that has an effect (as expected).

Data and the complete list of results are provided in an online repository.

## Final remarks

A commented R code for all the analyses discussed in this article can be found in an online repository (link to the url: https://github.com/akurz1/MixedLogit). It contains a function called estimation, which provides conditional maximum likelihood estimates (of baseline and slope parameters), standard errors, statistical tests, and information criteria. It depends on the package psychotools (Zeileis et al., 2023). It requires only two arguments, i.e., response matrix and covariate matrix (both must be numerical, no data frames or other R objects). In respect of the covariate matrix each column must contain the respective covariate values of the persons. Thus, it must have as many columns as covariates are considered. In the case of one covariate only, it must also be a matrix, i.e., a one-column matrix. Additionally, a file is provided with an R script for an analysis using the parameter-free Rao score test that depends on the package RaschSampler (Verhelst et al., 2007). At the moment both can only be used with complete data matrices, i.e., no missing values are allowed. Persons with missing values have to be excluded from the analysis. The authors currently work on an extension of the code in two respects: the consideration of missing values and a generalization of the approach to mixed effects logit models that consider responses in potentially more than two categories. This would, for instance, allow testing item parameter invariance in the partial credit model (Masters, 1982), which is a model for ordinal responses. Once this additional work is completed, the extended code will be included in the R package tcl (Draxler & Kurz, 2023) for the next update of the package.

The conditional likelihood approach involves computational issues that are particularly noteworthy. In the present context, the $\gamma$ functions depend on all the covariate values of a person. Thus, for persons with different covariate values one yields different $\gamma$ functions (except for rare special cases). The more covariates are considered in an application, and even more so when real-valued covariates are used, the less likely is it for two or more persons to obtain exactly the same covariate values. The R code, therefore, computes the $\gamma$ functions for every single person in the sample separately which is, of course, computationally intensive. Nevertheless, computation times are certainly acceptable for typical sample sizes, i.e., up to a few thousand persons. For the examples presented in this article, it is only a matter of seconds. Thus, the approach does not seem to be of any substantial practical limitation. When considering only one or very few covariates, in particular, binary ones this code will be (rather) inefficient. The more persons are contained in the sample whose covariate values are exactly the same, the more inefficient is it to compute their $\gamma$ functions separately, of course.

The main objective of this article relates to a typical psychometric problem. It discusses a mixed-effects logit model and an inferential approach of measurement or item parameter invariance for multiple (potentially real-valued) covariates simultaneously. Thus, it avoids carrying out multiple statistical tests and the accumulation of respective error probabilities. An additional aim of this article is to provide an incentive for researchers to apply such a mixed-effects model in longitudinal designs and to investigate effects of covariates or predictors or explanatory variables over a period of time (or different experimental conditions) as illustrated in Example 3. Such applications of mixed-effects models for binary data, even though dating back to the work of Cox (1958), are not quite well-known and, thus, rather seldom in behavioral research.

At the core of this work is the conditional maximum likelihood approach. It eliminates random effects (i.e., person parameters) and, in case of conditioning on the observed values of both row and column sums of the response matrix, also effects of other nuisance parameters (i.e., $\alpha$ parameters). This approach has a long tradition, in particular, in the Rasch modeling framework. It implies that the item parameters are estimated independently of the person parameters. Furthermore, by eliminating random effects or the person parameters one also yields a solution of a technical problem related to the properties of the estimates discussed by Neyman and Scott (1948). Another solution that is also widely used in psychometric problems is the marginal maximum likelihood approach. Roughly speaking, it eliminates the effects of individual person parameters (i.e., random effects) by averaging over an assumed population. Thus, it comes at the cost of an assumption on the (unknown) distribution of the person parameters but, principally, it is straightforwardly applicable in the present problem too.

The last remark on the concept of conditioning is of a deeper theoretical and philosophical nature and involves the following argument. Different schools of statistical inference (in particular, frequentist and Bayesian) only agree on the problem of conditioning when the statistic is ancillary (a notion that goes back to R.A. Fisher), i.e., when its probability distribution does not depend on the parameters of interest. This is, of course, not the case in the present problem. The distributions of the row and column sums of the response matrix $\varvec{R}$ and $\varvec{S}$ do depend on all parameters of the model. A further extensive discussion on the conditional approach, particularly in reference to psychometric problems has been given by Skrondal and Raabe-Hesketh (2022).

## Data Availability

Data examples and results of the analysis are available in an online repository. Link to the url: https://github.com/akurz1/MixedLogit

## Appendix A: Technical details on score function and information matrix

The vector-valued score function is given by

$$ \varvec{A}(s_2, \dots , s_k, \varvec{\alpha }, \varvec{\delta }) = \begin{pmatrix} \frac{\partial \ell (\varvec{\alpha }, \varvec{\delta })}{\partial \alpha _2}\\ \vdots \\ \frac{\partial \ell (\varvec{\alpha }, \varvec{\delta })}{\partial \alpha _k} \end{pmatrix} = \begin{pmatrix} s_2 - E(S_2)\\ \vdots \\ s_k - E(S_k) \end{pmatrix} = \begin{pmatrix} s_2 - \sum _i \gamma _r^{-1} \frac{\partial \gamma _r}{\partial \alpha _2}\\ \vdots \\ s_k - \sum _i \gamma _r^{-1} \frac{\partial \gamma _r}{\partial \alpha _k} \end{pmatrix}, $$

$$\begin{aligned} \varvec{D}_p(t_{2p}, \dots , t_{kp}, \varvec{\alpha }, \varvec{\delta }) = \begin{pmatrix} \frac{\partial \ell (\varvec{\alpha }, \varvec{\delta })}{\partial \delta _{2p}}\\ \vdots \\ \frac{\partial \ell (\varvec{\alpha }, \varvec{\delta })}{\partial \delta _{kp}} \end{pmatrix} = \begin{pmatrix} t_{2p} - E(T_{2p})\\ \vdots \\ t_{kp} - E(T_{kp}) \end{pmatrix} \\ = \begin{pmatrix} t_{2p} - \sum _i \gamma _r^{-1} \frac{\partial \gamma _r}{\partial \delta _{2p}}\\ \vdots \\ t_{kp} - \sum _i \gamma _r^{-1} \frac{\partial \gamma _r}{\partial \delta _{kp}} \end{pmatrix} \hspace{0.1cm} \forall \textit{p}, \end{aligned}$$

$$ \varvec{D}(s_2, \dots , s_k, t_{21}, \dots , t_{k1}, \dots , t_{2q}, \dots , t_{kq}, \varvec{\alpha }, \varvec{\delta }) = \begin{pmatrix} \varvec{A}\\ \varvec{D}_1\\ \vdots \\ \varvec{D}_q \end{pmatrix}. $$

Note that all expected values are conditional on $\varvec{R} = \varvec{r}$.

The Fisher information matrix is the covariance of the score, i.e.,

$$ \varvec{F}(\varvec{\alpha }, \varvec{\delta }) = Cov \big (\varvec{D} \big ) = E \big (\varvec{D} \varvec{D}^{\top } \big ). $$

It is a positive-definite square matrix of order $(k - 1) (q + 1)$ (i.e., the number of free parameters) and can be obtained by

$$ \varvec{F}(\varvec{\alpha }, \varvec{\delta }) = -E \Bigg (\frac{\partial ^2 \ell (\varvec{\alpha }, \varvec{\delta })}{\partial (\varvec{\alpha }_*^{\top }, \varvec{\delta }_*^{\top })^{\top } \partial (\varvec{\alpha }_*^{\top }, \varvec{\delta }_*^{\top })} \Bigg ). $$

The R code provided in an online repository uses the following reparameterization to compute the Fisher information matrix. Let $\varvec{\beta }_i: \mathbb {R}^{k(q + 1)} \rightarrow \mathbb {R}^k$,

$$ \varvec{\beta }_i(\varvec{\alpha }, \varvec{\delta }, x_{i1}, \dots , x_{iq}) = \begin{pmatrix} \beta _{i1}\\ \vdots \\ \beta _{ik} \end{pmatrix} = \begin{pmatrix} \alpha _1 + \sum _{p = 1}^q\delta _{1p} x_{ip}\\ \vdots \\ \alpha _k + \sum _{p = 1}^q\delta _{kp} x_{ip} \end{pmatrix} \forall i $$

denote a *k*-vector of person-specific item parameters. Thus, potentially all persons can have different item parameters depending on their covariate values. Let $\varvec{\beta }^{\top } = (\varvec{\beta }_1^{\top }, \dots , \varvec{\beta }_n^{\top })$ and

$$ \varvec{J} = \frac{\partial \varvec{\beta }}{\partial (\varvec{\alpha }_*^{\top }, \varvec{\delta }_*^{\top })} $$

be its Jacobian, i.e., a $nk \times (k - 1)(q + 1)$ matrix of first order partial derivatives with respect to all free parameters $\varvec{\alpha }_*^{\top } = (\alpha _2, \dots , \alpha _k)$ and $\varvec{\delta }_*^{\top } = (\varvec{\delta }_{1*}^{\top }, \dots , \varvec{\delta }_{q*}^{\top }), \hspace{0.1cm} \varvec{\delta }_{p*}^{\top } = (\delta _{2p}, \dots , \delta _{kp})$. Then, the Fisher information matrix is obtained by

$$ \varvec{F}(\varvec{\alpha }, \varvec{\delta }) = \varvec{J}^{\top } \varvec{F}(\varvec{\beta }) \varvec{J}, $$

where $\varvec{F}(\varvec{\beta })$ is the information matrix for the reparameterized case of person-specific item parameters. It is a block-diagonal matrix with *n* blocks each one of order *k*. Thus, a block represents the contribution (of information) of a single person. The entries of the *i*th block on the diagonal are given by

$$ \gamma _{r_i}^{-1} \frac{\partial \gamma _{r_i}}{\partial \beta _{ij}} \Bigg (1 - \gamma _{r_i}^{-1} \frac{\partial \gamma _{r_i}}{\partial \beta _{ij}} \Bigg ) \hspace{0.5cm} \forall j $$

and off-diagonal by

$$ \gamma _{r_i}^{-1} \frac{\partial ^2 \gamma _{r_i}}{\partial \beta _{ij} \partial \beta _{il}} - \gamma _{r_i}^{-2} \frac{\partial \gamma _{r_i}}{\partial \beta _{ij}} \frac{\partial \gamma _{r_i}}{\partial \beta _{il}} \hspace{0.5cm} \forall j \ne l \hspace{0.5cm} l = 1,\dots , k, $$

where the $\gamma$ functions have also to be reparameterized accordingly, i.e.,

$$\begin{aligned}&\gamma _0(\varvec{\beta }_i) = 1\\&\gamma _1(\varvec{\beta }_i) = \exp (\beta _{i1}) + \dots + \exp (\beta _{ik})\\&\gamma _2(\varvec{\beta }_i) = \exp (\beta _{i1} + \beta _{i2}) + \exp (\beta _{i1} + \beta _{i3}) + \dots \\&\qquad \qquad \quad + \exp (\beta _{i,k - 1} + \beta _{ik})\\&\gamma _3(\varvec{\beta }_i) = \exp (\beta _{i1} + \beta _{i2} + \beta _{i3}) + \exp (\beta _{i1} + \beta _{i2} + \beta _{i4}) \\&\qquad \qquad \quad + \dots + \exp (\beta _{i,k - 2} + \beta _{i,k - 1} + \beta _{ik})\\&\vdots \\&\gamma _{k - 1}(\varvec{\beta }_i) = \exp (\beta _{i1} + \dots + \beta _{i,k - 1}) + \dots \\&\qquad \qquad \qquad + \exp (\beta _{i2} + \dots + \beta _{ik})\\&\gamma _k(\varvec{\beta }_i) = \exp (\beta _{i1} + \dots + \beta _{ik}) \end{aligned}$$

$$\forall i$$ and with $$\beta _{ij} = \alpha _j + \sum _p \delta _{jp} x_{ip}$$.

## Appendix B: The case of conditioning on both *R = r* and *S = s*

In case of conditioning on both $\varvec{R} = \varvec{r}$ and $\varvec{S} = \varvec{s}$ the CML estimate of $\varvec{\delta }$ is obtained by

$$ \widehat{\varvec{\delta }} := \underset{\varvec{\delta }\in \mathbb {R}^{kq}}{\arg \max } \hspace{0.2cm} \ell (\varvec{\delta }). $$

Given mild conditions from general likelihood theory and the conditional case (Andersen, 1970; Pfanzagl, 1993) it holds that

$$ \widehat{\varvec{\delta }}_* \xrightarrow {P} \varvec{\delta }_* $$

and

$$ \sqrt{n} \big (\widehat{\varvec{\delta }}_* - \varvec{\delta }_* \big ) \xrightarrow {D} N \big ( \varvec{0}_{(k - 1)q}, \varvec{F}^{-1}(\varvec{\delta }) \big ), $$

when $n \rightarrow \infty$, where $\varvec{F}^{-1}(\varvec{\delta })$ denotes the asymptotic covariance matrix of $\varvec{\delta }_*^{\top } = (\varvec{\delta }_{1*}^{\top }, \dots , \varvec{\delta }_{q*}^{\top }), \, \varvec{\delta }_{p*}^{\top } = (\delta _{2p}, \dots , \delta _{kp})$.

The vector-valued score function, i.e., a $(k - 1)q \times 1$ matrix, is given by

$$ \varvec{D}_p(t_{2p}, \dots , t_{kp}, \varvec{\delta }) = \begin{pmatrix} \frac{\partial \ell (\varvec{\delta })}{\partial \delta _{2p}}\\ \vdots \\ \frac{\partial \ell (\varvec{\delta })}{\partial \delta _{kp}} \end{pmatrix} \forall \textit{p}, $$

$$ \varvec{D}(s_2, \dots , s_k, t_{21}, \dots , t_{k1}, \dots , t_{2q}, \dots , t_{kq}, \varvec{\delta }) = \begin{pmatrix} \varvec{D}_1\\ \vdots \\ \varvec{D}_q \end{pmatrix}. $$

and the Fisher information matrix by

$$ \varvec{F}(\varvec{\delta }) = -E \Bigg (\frac{\partial ^2 \ell (\varvec{\delta })}{\partial \varvec{\delta }_* \partial \varvec{\delta }_*^{\top }} \Bigg ). $$

The four test statistics based on asymptotic theory are then obtained by

$$ LR = -2 \big (\ell (\varvec{\delta }= \varvec{0}) - \ell (\widehat{\varvec{\delta }}) \big ), $$

$$ RS = \varvec{D}^{\top }(\varvec{\delta }= \varvec{0}) \varvec{F}^{-1}(\varvec{\delta }= \varvec{0}) \varvec{D}(\varvec{\delta }= \varvec{0}), $$

$$ W = \widehat{\varvec{\delta }}_*^{\top } \varvec{F}(\widehat{\varvec{\delta }}) \widehat{\varvec{\delta }}_*, $$

$$ GR = \varvec{D}^{\top }(\varvec{\delta }= \varvec{0}) \widehat{\varvec{\delta }}_*. $$

When $(\varvec{\tau }, \varvec{\alpha }, \varvec{\delta }) \in \Theta _1$ is true and $n \rightarrow \infty$ their common limiting distribution is the central $\chi ^2$ with $df = (k - 1)q$.

## Appendix C: Additional results from Examples 1 and 2, Sec. “Example 1” and “Example 2”

**Table 9:** *Z*-test statistics referring to parameters of Table 1

	Math data				Reading data			
Item	Baseline	Gender	Hisei	Migra	Baseline	Gender	Hisei	Migra
2	$-0.654$	0.511	$-1.300$	$-0.165$	$-4.349$	$-1.311$	$-0.809$	0.606
3	$-4.215$	$-0.478$	$-1.895$	$-1.034$	$-15.250$	$-1.579$	$-0.920$	0.142
4	6.076	1.252	$-1.995$	0.536	4.849	0.321	0.125	0.481
5	1.332	1.610	$-0.655$	0.304	$-0.736$	$-1.820$	$-0.502$	1.472
6	5.454	0.979	0.279	1.362	0.378	$-1.181$	1.778	0.251
7	0.026	1.835	$-2.505$	1.490	$-6.875$	$-1.783$	$-1.717$	0.004
8	$-0.031$	2.225	$-0.791$	1.253	$-0.058$	$-0.913$	$-0.991$	$-0.150$
9	$-1.281$	4.909	1.094	0.135	$-7.501$	$-2.360$	$-0.803$	0.607
10	0.448	2.739	$-1.007$	0.881	$-9.602$	$-2.617$	$-1.910$	$-0.029$
11	$-3.027$	4.442	0.285	1.417	$-14.275$	$-3.042$	$-2.599$	$-0.200$
12	$-$	$-$	$-$	$-$	$-1.556$	$-3.122$	0.077	0.474

Note. Item 1 omitted. Its parameters are set to 0 for identifiability

**Table 10:** The corresponding *p* values of Table 9

	Math data				Reading data			
Item	Baseline	Gender	Hisei	Migra	Baseline	Gender	Hisei	Migra
2	0.513	0.609	0.194	0.869	$<0.001$	0.190	0.418	0.544
3	$<0.001$	0.633	0.058	0.301	$<0.001$	0.114	0.358	0.887
4	$<0.001$	0.211	0.046	0.592	$<0.001$	0.748	0.900	0.631
5	0.183	0.107	0.512	0.761	0.461	0.069	0.615	0.141
6	$<0.001$	0.327	0.780	0.173	0.705	0.237	0.075	0.802
7	0.980	0.066	0.012	0.136	$<0.001$	0.075	0.086	0.997
8	0.975	0.026	0.429	0.210	0.954	0.361	0.322	0.880
9	0.200	$<0.001$	0.274	0.893	$<0.001$	0.018	0.422	0.544
10	0.654	0.006	0.314	0.378	$<0.001$	0.009	0.056	0.977
11	0.002	$<0.001$	0.776	0.156	$<0.001$	0.002	0.009	0.841
12	$-$	$-$	$-$	$-$	0.120	0.002	0.939	0.635

Note. Item 1 omitted. Its parameters are set to 0 for identifiability

**Table 11:** *Z*-test statistics referring to parameters of Table 4 with corresponding *p* values in parentheses

Item	Base	Gender	Age	Mean grade
2	$-$0.454 (0.650)	0.425 (0.671)	0.437 (0.662)	$-$0.534 (0.593)
3	0.228 (0.820)	0.419 (0.675)	$-$0.319 (0.750)	$-$0.791 (0.429)
4	$-$0.177 (0.859)	0.149 (0.881)	$-$0.371 (0.711)	$-$0.689 (0.491)
5	$-$0.920 (0.357)	1.173 (0.241)	0.100 (0.920)	$-$0.881 (0.378)
6	$-$0.750 (0.453)	1.130 (0.258)	0.309 (0.757)	$-$0.725 (0.468)
7	$-$0.526 (0.599)	0.611 (0.541)	$-$0.444 (0.657)	$-$0.797 (0.426)
8	0.076 (0.940)	0.158 (0.874)	0.000 (1.000)	$-$0.942 (0.346)
9	$-$1.112 (0.266)	0.125 (0.901)	0.919 (0.358)	$-$0.193 (0.847)
10	$-$0.688 (0.491)	0.770 (0.441)	$-$0.426 (0.670)	$-$0.458 (0.647)

Note. Item 1 omitted. Its parameters are set to 0 for identifiability

## Code availability

R codes for all computations discussed in this research are available in an online repository. Link to the url: https://github.com/akurz1/MixedLogit
